# Editorial: Epithelial-mesenchymal transition (EMT) as a therapeutic target in cancer, Volume II

**DOI:** 10.3389/fonc.2023.1218855

**Published:** 2023-06-20

**Authors:** Weilong Zhong, Tao Sun

**Affiliations:** ^1^ Department of Gastroenterology and Hepatology, General Hospital, Tianjin Institute of Digestive Diseases, Tianjin Key Laboratory of Digestive Diseases, Tianjin Medical University, Tianjin, China; ^2^ State Key Laboratory of Medicinal Chemical Biology and College of Pharmacy, Nankai University, Tianjin, China

**Keywords:** epithelial-mesenchymal transition, signaling pathway, anticancer therapy, pharmacological research, therapeutic target

Epithelial-mesenchymal transition (EMT) is a biological process that refers to the transformation of epithelial cells into mesenchymal cells (1). During this process, changes occur in cell morphology, cell-cell interactions, and matrix adhesion, which give cells stronger migration and invasion abilities (2). EMT plays an important role in physiological and pathological processes such as embryonic development, tissue repair, and cancer metastasis (3). In cancer, EMT is considered one of the important mechanisms of tumor metastasis and drug resistance (4–6). EMT causes cancer cells to detach from their original site and enter the blood or lymphatic system, forming metastatic foci in other parts of the body. In addition, EMT also makes cancer cells resistant to treatment, making treatment more difficult (7). Therefore, EMT has become an important target for cancer treatment (8, 9). Currently, there are two main strategies for targeting EMT: one is to inhibit the EMT process to prevent cancer cell metastasis and invasion. The other is to reverse the EMT process to restore the epithelial characteristics of cancer cells, making them more sensitive to treatment.

## 1 Strategies to suppress EMT process mainly include the following aspects

### 1.1 Targeting EMT transcription factors

EMT transcription factors are key regulatory factors in the EMT process, including Snail, Slug, Twist, etc. By targeting these transcription factors, EMT process can be inhibited, thereby preventing cancer cell metastasis and invasion.

### 1.2 Targeting EMT-related proteins

The EMT process also involves various proteins, including E-cadherin, N-cadherin, Vimentin, etc. By targeting these proteins, EMT process can be inhibited, thereby preventing cancer cell metastasis and invasion.

### 1.3 Targeting EMT-related signaling pathways

EMT process involves multiple signaling pathways, including Wnt, TGF-β, Notch, etc. By targeting these signaling pathways, EMT process can be inhibited, thereby preventing cancer cell metastasis and invasion. In an article published in this Research Topic, Cheng et al. reported the mechanism of RNF6 activating the TGF-β1/c-Myb pathway to promote EMT in esophageal squamous cell carcinoma. Cheng et al. found that upregulation of RNF6 can promote the progression of esophageal cancer, indicating poor prognosis. RNF6 also enhances the migration and invasion ability of ESCC cells *in vitro*. Silencing of RNF6 inhibits the migration and invasion of ESCC cells. The TGF-β inhibitor reverses the oncogenic effect of RNF6. RNF6 regulates the migration and invasion of ESCC cells by activating the TGF-β pathway. RNF6/TGF-β1 promotes the progression of esophageal cancer through c-Myb. RNF6 may promote the proliferation, invasion, and migration of ESCC cells and affect the progression of ESCC by activating the TGF-β1/c-Myb pathway.

In Liu et al.’s study, the combination of network pharmacology prediction and experimental validation showed that Ginkgolide K (GK) inhibits the invasion and metastasis of human lung adenocarcinoma cells through the Akt/GSK-3β/Snail and Wnt/β-catenin pathways. Lung cancer is one of the most common malignant tumors, with high mortality rates and increasing numbers of new cases worldwide. Ginkgo leaves have been used for the treatment of lung cancer for many years. Ginkgolide K is an important active ingredient extracted from Ginkgo. However, the mechanism by which Ginkgolide K inhibits the invasive and metastatic properties of lung cancer is not clear. The authors used network pharmacology methods to study the molecular mechanism of Ginkgolide K in inhibiting lung cancer metastasis. Then, potential target proteins between Ginkgolide K and lung cancer were analyzed. Finally, molecular docking and experimental validation were performed. The study found that there were 79 common genes involved in cell migration positively regulated by the cross-talk between lung cancer and Ginkgolide K. *In vitro* experiments showed that GK had a significant inhibitory effect on the invasion and metastasis of A549 and H1299 cells. In animal experiments, GK had a significant inhibitory effect on the metastasis of LLC. The experiment confirmed that GK can inhibit the Akt/GSK-3β/Snail and Wnt/β-catenin cascades in A549, H1299, and LLC cells, preventing metastasis. The results of this study are consistent with the hypothesis of network pharmacology analysis.

## 2 The strategies to reverse the EMT process mainly include the following aspects

### 2.1 Targeting EMT-related signaling pathways

By targeting EMT-related signaling pathways, the EMT process can be reversed, thereby restoring the epithelial characteristics of cancer cells and making them more sensitive to treatment. Xi et al. found that ACT001, a novel PAI-1 inhibitor, exerts a synergistic effect with cisplatin by inhibiting the PI3K/AKT pathway in glioblastoma (10). PAI-1 plays an important role in the occurrence, recurrence, and multidrug resistance of tumors and is highly expressed in tumors. ACT001 is currently in phase III clinical trials for the treatment of glioblastoma (GBM). However, the specific molecular mechanism of ACT001 is not clear. In this study, Xi et al. investigated the effect of ACT001 on the proliferation of glioblastoma cells and elucidated its mechanism. They found that ACT001 directly binds to PAI-1 to inhibit the PI3K/AKT pathway, thereby inducing the inhibition of glioblastoma cell proliferation, invasion, and migration. In addition, the combination of ACT001 and cisplatin showed a synergistic inhibitory effect on glioblastoma *in vitro* and *in vivo*.

In addition, Qiao and Tian reported that ATL-1 inhibits EMT by targeting Hsp27 and enhances the anti-tumor effect of cabozantinib in prostate cancer. MTT experiments showed that compared with the control group, ATL-1 had an inhibitory effect on the proliferation of prostate cancer cells DU145 and PC-3. TUNEL results showed that compared with the control group, silencing Hsp27 and ATL-1 treatment significantly promoted apoptosis of DU145 and PC-3 prostate cancer cells. qRT-PCR results showed that compared with the control group, ATL-1 promoted the expression of caspase-3, PARP, and Bax in DU145 and PC-3 prostate cancer cells. ATL-1 inhibits Hsp27, reduces cell viability, and induces cell apoptosis. ATL-1 inhibits Hsp27 to enhance the anti-tumor effect of cabozantinib. Hsp27 regulates eIF4E and mediates cell protection. ATL-1 can inhibit the malignant evolution of prostate cancer cells by inhibiting Hsp27/eIF4E. ATL-1 also enhances the chemosensitivity of cabozantinib in prostate cancer.

### 2.2 Targeting EMT-related proteins

By targeting EMT-related proteins, the EMT process can be reversed, thereby restoring the epithelial characteristics of cancer cells and making them more sensitive to treatment. Zhong et al. found that cartilage oligomeric matrix protein (COMP) promotes epithelial-mesenchymal transition (EMT) in colorectal cancer by interacting with Transgelin (11). The study found that COMP interacts with TAGLN in colorectal cancer EMT, regulates cell cytoskeleton remodeling, and promotes malignant progression. COMP is highly expressed in high-grade colorectal cancer and is positively correlated with TAGLN expression. Knockdown of COMP can inhibit the metastasis and invasion of colorectal cancer, while overexpression of COMP can promote EMT in colorectal cancer. Through virtual screening of protein interaction interfaces, the flavonoid compound chrysin was found to have the highest docking score with the COMP/TAGLN complex. Chrysin inhibits COMP, thereby preventing EMT and malignant progression of colorectal cancer. This study elucidates the role of COMP in EMT and suggests that COMP/TAGLN may be a potential therapeutic target for tumors. Chrysin has significant anti-tumor effects. This study provides a preliminary anti-tumor treatment method for inhibiting EMT by targeting COMP or its interacting proteins.

## 3 Conclusion

EMT is of great significance as a therapeutic target for cancer. By inhibiting the EMT process or reversing the EMT process, the metastasis and invasion of cancer cells can be prevented, thus improving the therapeutic effect.

## Author contributions

All authors listed have made a substantial, direct, and intellectual contribution to the work and approved it for publication.
